# Microglia networks within the tapestry of alzheimer’s disease through spatial transcriptomics

**DOI:** 10.1186/s13024-025-00897-y

**Published:** 2025-09-29

**Authors:** Yi Zhou, Christopher K. Glass

**Affiliations:** Department of Cellular and Molecular Medicine, University of California, San Diego, La Jolla, San Diego, CA USA

**Keywords:** Spatial transcriptomics, Alzheimer’s disease, Aging

## Abstract

Understanding Alzheimer’s disease (AD) at the cellular level requires insights into how diverse cell types respond to hallmark pathologies, including amyloid plaques and tau aggregates. Although single-cell transcriptomic approaches have illuminated the trajectories of AD progression in both animal models and human brains, they often lack the spatial context necessary to fully comprehend cell–cell interactions and microenvironmental influences. In this review, we discuss recent advances in spatial transcriptomics—integrating both imaging- and sequencing-based methods—that map gene expression within intact brain tissues. We highlight how these technologies have revealed regional heterogeneity and functional diversity among microglia, and their dynamic interactions with astrocytes, neurons, and oligodendrocytes in both aging and AD. Emphasis is placed on the interactions of microglia within the amyloid plaque niche, their contribution to synaptic degeneration, and how aging accelerates microglial and glial activation. By synthesizing findings from AD mouse models and physiologically characterized human tissues, we provide a comprehensive view of the cellular interplay driving AD pathogenesis and offer insights into potential therapeutic avenues.

## Background

Alzheimer’s disease (AD) is a progressive neurodegenerative disease most often associated with memory deficits and cognitive decline. The primary pathological hallmarks of AD include amyloid (Aβ) plaques, neurofibrillary tangles (NFTs), gliosis, and neuronal loss [1–5]; along with cerebrovascular amyloidosis, inflammation, and significant synaptic alterations [6–8]. In both mouse AD pathology and human AD brains, specific cell populations have been identified relative to amyloid plaque deposition, Braak stages, and cognitive function. These findings reveal a spectrum of selective vulnerability or resilience in neurodegeneration and provide insights into fundamental gene regulatory and epigenomic networks that are altered in AD [9, 10].

Although microgliosis and its association with distinct microglia phenotypes were first observed over a century ago, microglia have only recently emerged as a major focus in AD research [11, 12]. Genome-wide association studies (GWAS) have uncovered numerous common risk alleles that are likely to influence disease phenotypes by altering gene expression in microglia [13–17]. Single-cell RNA sequencing (scRNAseq) has further revealed diverse microglia states, linking their multifaceted functional roles to neuroinflammation and neurodegeneration [18–23]. However, a fundamental question remains unsolved: how do microglia and their neighborhood interact with the pathological hallmarks in the AD brains.

The maintenance of brain tissue integrity and homeostasis requires complex, coordinated activities of thousands of genes, which are expressed in hundreds of neuronal and non-neuronal cell types with specific spatial distribution patterns and distinct structural and functional properties. At the same time, the complex microenvironment composed of extracellular molecules, support matrices, and neighboring cells collectively shapes the phenotypic states of cells [24]. Although scRNA-seq continues to expand the understanding of cell states across a wide range of organisms and tissues, it fails to capture the context of microenvironments that nurture specific cell identities. Spatially resolved transcriptomic technology (ST) has emerged as a method to simultaneously measure transcripts at single-cell or near single-cell resolution in a multiplexed manner. By expanding the scope of single-cell biology from isolated cells to multicellular interaction in neighborhoods, ST has revealed recurrent and functional organizations of cells in different tissues and conditions [25–27].

In this review, we highlight recent advances and ongoing challenges in spatial transcriptomics (ST), providing insights into selecting appropriate ST tools and platforms for Alzheimer’s disease (AD) research. We focus particularly on recent biological findings involving microglia and their non-autonomous roles in communicating with other cell types within the AD brain. Additionally, we explore the complex microglial cellular networks—like threads intricately woven throughout the brain—that influence Alzheimer’s disease progression and aging.

### Overview of spatial transcriptomics technologies

**Fig. 1:**
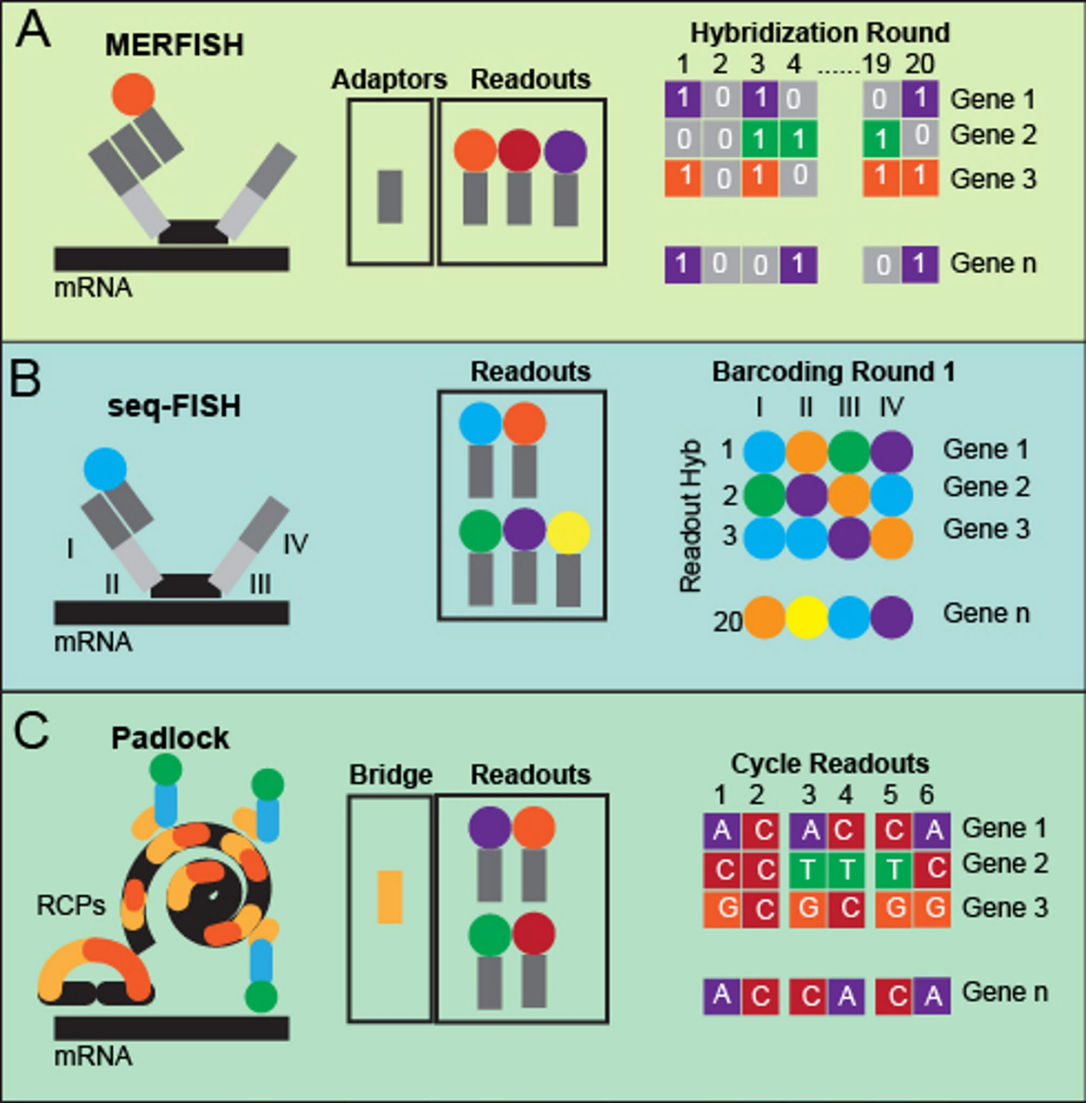
Hybridization and de-coding scheme of img-ST. **(A)** The encoding probes binding to target mRNA, signal detection, and decoding method for MERFISH with hemming distance 4 barcoding system. **(B)** The encoding probes bind to target mRNA, signal detection, and decoding method for seq-FISH with combinations of different fluorophores of readout probes. **(C)** The Padlock encoding probes bind to target mRNA, RCP, and signal detection with 2 to 6 nucleotides per cycle

For decades researchers have employed microscopy to obtain spatial and quantitative information on tissues. They have relied on targeted-protein-based techniques, such as immunohistochemistry (IHC) and immunofluorescence (IF), as well as on hybridization methods using complementary RNA or DNA probes, such as fluorescence in situ hybridization (FISH). In parallel, to achieve a genome-wide understanding of transcriptomics, next-generation sequencing —including bulk RNA sequencing and scRNAseq—has been used to profile transcriptome enrichment in cell populations. The urge to address questions about cell compositions, genome-wide transcriptional regulation, and cell-cell interactions, combined with advancements in DNA sequencing, oligonucleotide synthesis, and fluorescence microscopy, has driven the development of ST. In general, ST can be broadly classified into imaging-based (img-ST) and sequencing-based (seq-ST) approaches [28–30].

img-ST methods are built upon the foundation of FISH, which has long been refined to detect individual mRNA molecules at subcellular resolution [31]. FISH provides the spatial distribution and quantitative levels of mRNAs in cells, as well as information of mRNA dynamics [32, 33]. Traditional FISH, however, is limited in multiplexing capacity due to spectral overlap among fluorophores [34]. To overcome these constraints, img-ST approaches incorporate combinatorial barcoding strategies into probes and employ multiple rounds of imaging (Fig. 1).

For instance, MERFISH (Multiplexed Error-Robust FISH) assigns a unique combinatorial barcode to each mRNA species and is designed to ensure a minimum Hamming distance of 4 between barcodes, thereby providing robust error correction across several rounds of hybridization [35] (Fig. 1A). The MERFISH methodology is commercialized as MERSOCPE by Vizgen. Another method seqFISH achieves multiplexing by both encoding barcodes, and multiple readout probes detected through different fluorophores in successive imaging rounds. This is to generate a combinatorial “pseudocolor” readout that can identify thousands of transcripts [36, 37] (Fig. 1B). In addition to these hybridization-based methods, in situ sequencing (ISS) techniques have been developed that rely on padlock probes [38]. padlock probes hybridize cDNAs sequences by reverse transcription and become circularized; rolling-circle amplification (RCA) then generates discrete rolonies (localized amplified products) at the sites of hybridization (Fig. 1C). Sequencing-by-ligation chemistry is subsequently used to decode the barcode sequences with near single-nucleotide resolution [39]. More advanced versions of ISS have emerged, and commercialized platforms like 10x Genomics’ Xenium being a notable example [40, 41]. Another ISS method STARmap enables the mapping of thousands of mRNAs in tissue sections up to 150 μm thickness [42].

**Fig. 2:**
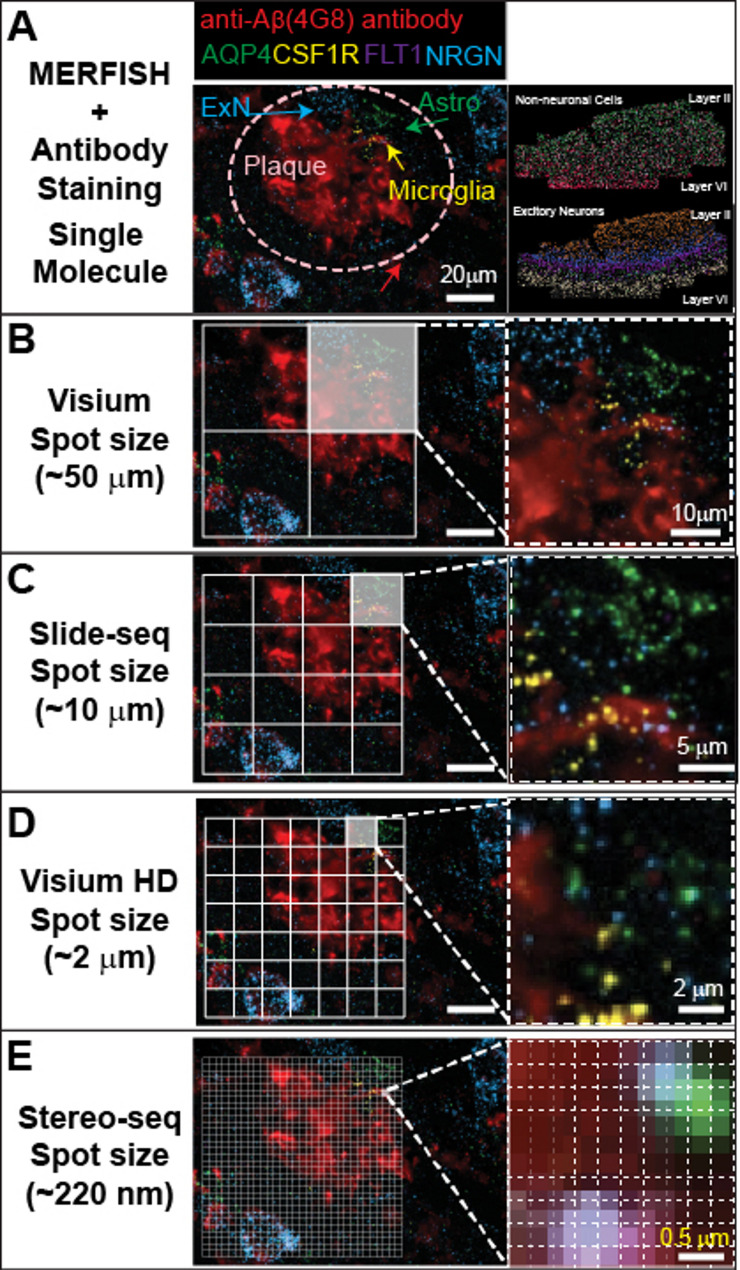
Resolution of representative img-ST and seq-ST technologies. **A.** Example image of unpublished img-ST MERFISH (~ 120 genes panel) data in human AD brain at single molecule level with anti-Aß (4G8) antibody staining and spatial mapping of neuronal and non-neuronal cells in the right panel. **B-D.** Grids mapped onto the image in (**A**) represent the resolution provided by Visium, Slide-seq, and Visium HD platforms, with the right panels indicating cells and molecules from single-molecule imaging in (**A**). **E.** Grid size of 0.5 μm on the left panel, and single box region in the right panel representing the 220 nm resolution of Stereo-seq spot size

Img-ST typically involves extended hybridization periods, imaging time and complicated decoding process to resolve ‘pre-decided’ genes. The drawback of img-ST is the pre-selection of a gene-panel, but this method provides sub-cellular resolution images. When coupled with advanced microscopy techniques, such as confocal or expansion microscopy, img-ST can achieve higher resolution and detect an extensive number of mRNA species. Advances in oligonucleotides synthesis and florescence labeling further enhance the flexibility and multiplexing capabilities of these technologies [28] (Fig. 2A). For instance, platforms using MERFISH method have demonstrated the imaging of over 2000 mRNA species imaging, and some reports suggest that similar systems like CosMx developed by NanoString may resolve between 2,000 and 6,000 RNA species. Moreover, img-ST can be combined with antibody-based protein staining on the same sample, enabling the simultaneous detection of RNA and proteins. This multimodal approach—especially when integrated with single-cell RNA sequencing—facilitates the identification of differentially expressed genes in intact brain environment with AD hall marks.

Seq-ST approaches utilize spatially barcoded oligonucleotide arrays to capture the entire transcriptome from mounted tissue sections. The pioneering concept was introduced by Stahl and colleagues [43]who immobilized oligonucleotides targeting all expressed genes on a chip. This foundational method evolved into platforms such as 10x Visium, which uses pre-defined capture spots on slides to collect mRNA while preserving tissue architecture with a resolution of about 50 μm (Fig. 2B and D). To further enhance spatial resolution, Slide-seq was developed by depositing densely packed, DNA-barcoded beads onto a surface. These beads capture transcripts from the tissue above, enabling finer mapping of gene expression with the resolution of about 10 µm [44] (Fig. 2C). Additional innovations have broadened the seq-ST toolbox. For example, DBiT-seq (Deterministic Barcoding in Tissue for spatial omics sequencing) employs microfluidic channels to deliver barcodes directly onto tissue sections, enabling simultaneous profiling of multiple molecular layers at high spatial resolution [45]. An even higher resolution is achieved with Stereo-seq from BGI, which utilizes nanoball-DNA for mRNA detection and can resolve imaging spots down to approximately 220 nm [46] (Fig. 2E). Another noteworthy method is FISSEQ (Fluorescent In Situ Sequencing), which uses RCA to generate localized amplicons that are then sequenced using either sequencing-by-ligation or sequencing-by-synthesis chemistries. FISSEQ can achieve subcellular resolution (around 100–200 nm), though its sensitivity is limited to detecting approximately 100–200 transcripts per cell under standard experimental conditions [47]. In parallel, platforms like GeoMx integrate UV-cleavable oligonucleotides and oligo-conjugated antibodies to facilitate the spatially resolved detection of both RNA and protein, thereby bridging transcriptomic and proteomic analyses.

Collectively, current methods often involve trade-offs between transcriptome coverage, resolution of spatial context, and multi-module detection, underscoring the need for continued innovation in the field. In the context of AD research, ST has provided a powerful means to map gene expression and dysregulated molecule networks in AD brains and their association with pathogenic hallmarks.

### Microglia regional and functional heterogeneity

Compared to specific region-restricted neuronal cells, microglia are widely distributed throughout the brain, constituting approximately 4–10% of central nervous system cells [48]. Although traditionally considered a relatively homogeneous population, numerous studies have revealed significant regional heterogeneity in microglial density, morphology, and transcriptional profiles [28, 49–53]. For example, a brain-wide gradient pattern has been observed that microglia tend to be more abundant in rostral and dorsal regions and less abundant in ventral and caudal areas of the brain [49, 54]. Similar densities of microglia are found in the cortex, striatum, and hippocampus, whereas the thalamus and midbrain exhibit nearly half the density, and the cerebellum even lower.

Owing to their relatively small cell bodies, microglia can be readily isolated using fluorescence-activated cell sorting [55]. Subsequent scRNAseq of microglia isolated from different brain regions of the brain has uncovered the transcriptomic heterogeneity [56]. For example, microglia from the ‘deep brain’s basal ganglia (BG) showed specific anatomical features that potentially contributed to BG circuit function, and their regional variation mirrors the local astrocytes abundance [57]. Also, microglia in the cerebellum, but not in the striatum or cortex showed high levels of basal clearance activity which correlated with an elevated degree of cerebella neuronal attrition [58]. During development microglial transcriptomic clusters show regional-specific distributions; however, their responses to local cues become more pronounced in juvenile and adult mice [56, 59].

**Fig. 3:**
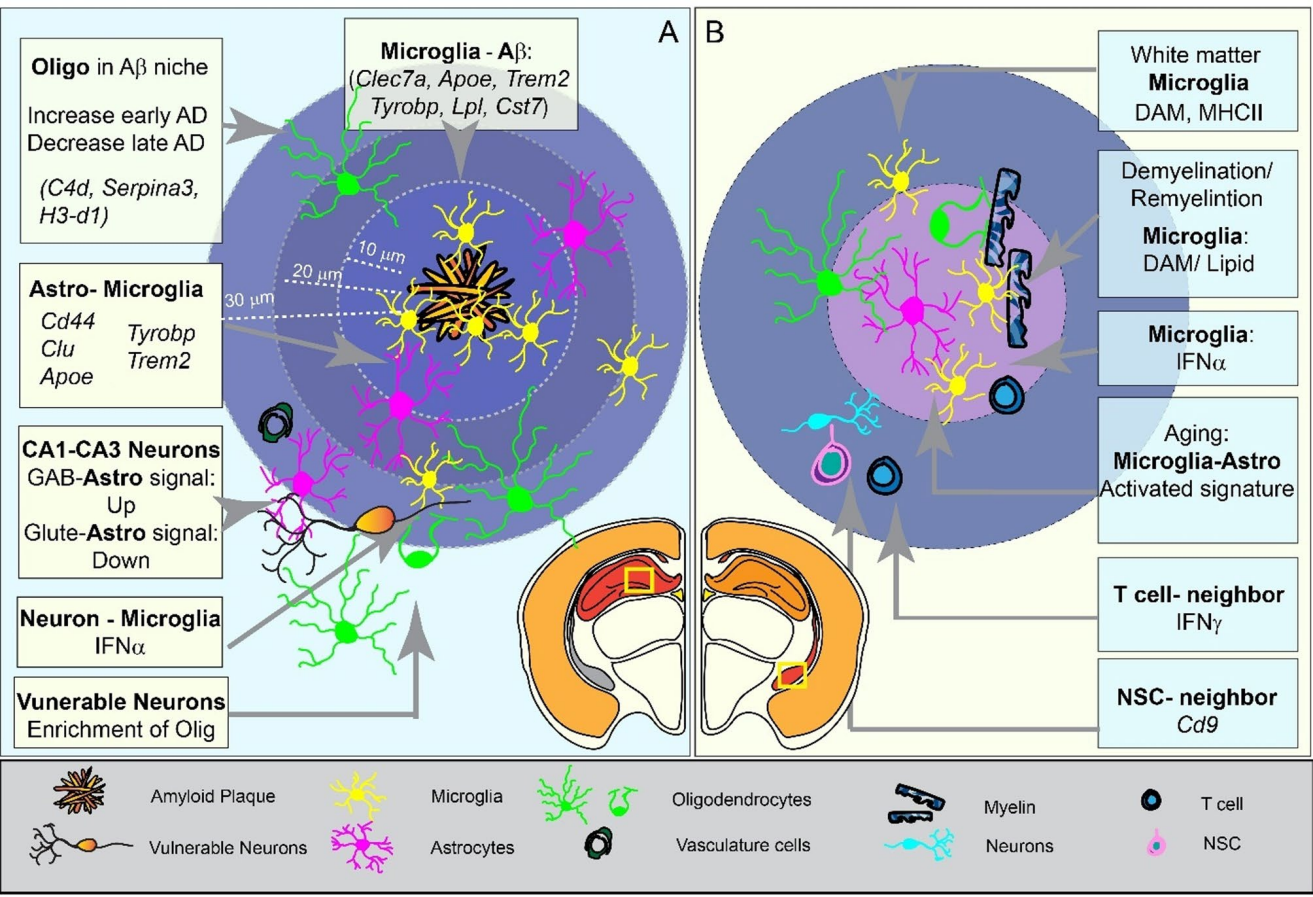
Cell-Cell communication in AD and aging mouse brain. **(A)** Cellular communications and interactions with Ab in AD mouse brain. **(B)** Cellular communications and interactions with myelin in aging mouse brain

Microglia exhibit a dynamic range of functional states, facilitated by a diverse set of cell surface markers that enable them to detect and respond to environmental changes [60]. DAMs (Disease associated microglia) were first identified in 5xFAD and APP/PS1 mice from scRNAseq of sorted microglia [61]. In addition, ARMs (Activated response microglia) have been characterized in AD pathology mouse model [62]while MGnD (microglial neurodegenerative phenotype) associated with neuritic dystrophy, has been observed in multiple neurodegeneration mouse models including those for amyotrophic lateral sclerosis (ALS) and multiple sclerosis (MS) [63]. Notably, ARMs and MGnDs share many similar signatures with DAMs. Following activation, microglia upregulate a suite of genes that define DAMs, such as *Clec7a*, *B2m*, *Apoe*, *Trem2*, and its adaptor *Tyrobp*, as well as genes involved in phagocytosis and lipid metabolism like *Cst7* and *Lpl* (Figure.3 A).

In the neurodegeneration models, such as the facial nerve axotomy model and demyelination pathogenesis model, significant microglial activation occurs in conjunction with the loss of oligodendrocytes in the corpus callosum [59]. In these contexts, genes including *Fam20c*, *Cst7*, *Ccl6*, *Fn1*, *Ank*, *Psat1*, and *Spp1* are enriched to variable degrees in demyelination associated microglia. Similar corresponding clusters have been identified in both healthy human brains and in the brains of patients with MS. Moreover a conserved microglial state marked by type I interferon (IFN)-responsive signature characterized by the enrichment of interferon-responsive genes (such as *Ifit3*, *Oasl2* and *Irf7*) has been observed across aging, neurodegeneration and other pathologies [56, 62, 64]. Collectively, scRNAseq data from regionally dissected and sorted microglia indicate that these cells adopt a spectrum of distinct functional states that are shaped by local environmental cues (Figure. 3B).

### Microglia in the amyloid plaque niche

A central question in AD research is how amyloid plaques relate to the neurodegenerative process [65–67]. Amyloid plaques may serve as initial triggers for AD [61], and genetic studies have linked sporadic AD risk to microglia expressed genes that are activated in response to amyloid deposition [62, 63, 68–72]. Microglia display an activated ‘ameboid’ morphology with larger cell bodies and fewer, retracted processes near amyloid plaques. In this activated state, microglia participate in amyloid deposition through endocytosis and contribute to the remodeling of plaque dense cores [73]. DAMs and MGnD are predominantly found surrounding plaques. For example, the DAM marker Lpl has been co-localized with microglial marker IBA1 and Thioflavin S staining for amyloid [69]while MGnD marker Clec7a, shows similar plaque-associated enrichment [6].

The advent of scRNAseq in AD brain tissue has revolutionized our understanding of cellular heterogeneity, revealing substantial and diverse changes in gene expression across microglia, astrocytes, neurons, and oligodendrocytes in both human and mouse AD models [74–77]. Moreover, astrocytes, neurons, and oligodendrocytes also display altered molecular responses to amyloid plaques [78]. However, to fully capture the spatial relationships among amyloid plaques, tau aggregates, cell death, and synapse loss—and to unravel the resulting complex cellular responses—ST has become an indispensable tool.

The first ST study in AD, conducted on both mouse brain and human brain samples, identified multi-cellular co-expressed gene networks associated with amyloid plaques, termed plaque-induced genes (PIGs) [79]. The plaque cellular niche was defined by expanding a 50 μm masking area of the Ab staining boundary. Using a seq-ST method of a spot imaging size of 100 μm in the *App*^*NL−G−F*^ mouse model, with adjacent brain tissue staining with Aß, 57 different PIGs were identified [43]. These PIGs shared a strong association with DAMs and inflammatory astrocytes. Notably, while the PIG response is primarily driven by microglia, several PIGs are expressed across multiple cell types confirmed by img-ST in situ sequencing methods [80]. For example, *Cyba* is present in both microglia and oligodendrocytes, and *Cd9* is expressed in astrocytes, microglia, and oligodendrocytes. Some inflammatory molecules (e.g., *H2-K1*, *Ly86*, and *Mpeg1*) and lysosomal enzymes (e.g., *Lgmn* and *Ctsa*) are detected in both microglia and neurons.

Another study utilizing img-ST (STARmap Plus) in the TauPS2APP mouse model that simultaneously profiled 2,766 RNA species alongside antibody staining for amyloid and phosphorated-tau [81]. Among the 13 major cell types, microglia, astrocytes, oligodendrocytes, oligodendrocyte precursor cells (OPC) and endothelial cells showed relative enrichment in the vicinity of plaques. Microglia were the most enriched population within the first 10 μm and 10–20 μm regions surrounding plaques, whereas other glia populations peaked in a 10–30 μm zone. Spatially Differential expression analysis in microglia revealed genes implicated in cell death regulation (e.g., *Ccl3*, *C1qa* and *Ctsd*) and regulation of cell migration (e.g., *Cd9*, *Apoe* and *Trem2*), which overlap with DAM signatures (Fig. 3A).

### Microglia’s neighbors in the amyloid plaque niche

Astrocytes are also key players in the amyloid plaque niche. Reactive astrocytes, such as disease-associated astrocytes (DAA), with characteristic gene signatures in AD patients and mouse models, implicated a major transcriptional response in AD to multiple cell types, particularly respond to microglia [75, 77, 82, 83]. In the cortex and hippocampus regions of TauPS2APP mice, DAA-like cells account for about 14% of all astrocyte populations, they are enriched around plaques at an intermediate distance (10–40 μm), and are marked by expression of *Gfab*, *Vim* and *Ctsb* genes [81]. Another study that utilized the img-ST platform from CosMx and seq-ST platform (Stereo-seq) further revealed a region-specific astrocytic response: hippocampal astrocytes near plaques show increased genes *Itm2b*, *Cpe*, and *C1qa*, while the cortical astrocytic response is characterized by *Atp1a1*, *Ckb*, and *Kcnip4*. Moreover, analysis of receptor-ligand (RL) interactions identified 130 candidate pairs between microglia and astrocytes, and 11 of them are PIGs. The strength of these interactions, including microglia-to-microglia signals (e.g., *Csf1* to *Csf1r* and *Cd44* to *Tyrobp*) and astrocyte-to-microglia crosstalk (e.g., astrocytic *Cd44*, *Clu*, *Apoe* with microglial *Tyrobp* and *Trem2*), increases as cells approaching plaques [84] (Fig. 3A).

Oligodendrocytes also respond to amyloid pathology [85]. In 5xFAD mouse brains, oligodendrocytes show a progressive transcriptional response with upregulation of genes such as *C4b*, *Serpina3n*, and *H3-d1*. Co-labeling of Ab and *Olig2* RNA revealed a significant increase in an oligodendrocyte specific plaque-associated signature [86]. Interestingly, regional factors rather than Aβ burden alone appear to drive oligodendrocyte gene expression: while oligodendrocyte modules are upregulated early (e.g., at 3 months in App^NL−G−F^ mice), they decrease within the immediate plaque niche. This suggests that an early protective response may eventually be overwhelmed as plaque load increases [79]. Additionally, STARmap Plus analyses have shown that distinct oligodendrocyte clusters are enriched 20–40 μm distance from plaques, and OPCs are notably enriched within 10–30 μm from plaques in late AD stages, indicating potential in situ proliferation and differentiation.

In contrast to the accumulation of glia, neuron density around plaques declined progressively [81]. Several PIGs are markedly increased in neurons, including lysosomal enzymes (*Lgmn*,* Ctsa*), regulators of lysosomal degradation (*Gns* and *Grn*), an inhibitor of Aβ aggregation (*Itm2b*), and a regulator of insulin growth factor (I*gfbp5*) expressed in neurons. *Serpina3* shifts its primary expression from neurons to astrocytes with disease progression [79]. In the hippocampal CA1-CA3 regions, GABA-signaling Receptor-Ligand pairs between astrocytes and CA1–CA3 and CA4 neurons (e.g., astrocytic *Gad1/2* + *Slc6a11* to neuronal *Gabbr2*) increase in strength as the plaque niches become more densely packed with microglia. In contrast, glutamate-signaling decreases (e.g., astrocytic *Gls* + *Slc1a3* to neuronal *Grik2*, *Grm8*, and *Gria4*). Interestingly, both GABA- and Glutamate-signaling from astrocytes to dentate gyrus (DG) neurons decreases, indicating a more complicated relationship between astrocytes and DG cells. Moreover, the upregulation of *Nav3*—an axon guidance regulator known to be increased in AD—in CA1–CA3 neurons near plaques underscores these multifaceted neuronal responses [84].

Additional insights have been provided by MERFISH studies in 5xFAD and *Trem2*^R47H^; 5xFAD brains where the TREM2 R47H variant in microglia significantly altered neuronal gene expression [87]. Another potential crosstalk mechanism between microglia and neurons in AD brain may be mediated by interferon pathways [88]. This study showed that half of the Clec7a ^+^ DAMs are Interferon responsive in 5xFAD mice. Deletion of microglial *Ifnar1* near plaques reduces post-synaptic terminal loss by mitigating selective engulfment, whereas neural *Ifnar1* deletion restored pre-synaptic terminals and decreased plaque accumulation. Overall, IFN-I signaling represents a critical module within the neuroinflammatory network of AD and prompts concerted cellular states that are detrimental to memory and cognition (Fig. 3A).

This emerging view provides evidence that the amyloid plaque niche is a highly dynamic environment where diverse cellular responses— predominantly mediated by microglia, but also involving astrocytes, oligodendrocytes, and neurons—contribute to the complex pathology of AD.

### Microglia and vulnerable neurons

The accumulation of misfolded tau aggregates within neurons is a defining feature of AD. In the human brain cortex, the earliest areas affected by AD pathology are the entorhinal cortex and transentorhinal cortex, followed by the hippocampal CA1 region [89] Several neuronal subtypes have been reported to be particularly vulnerable in AD [89–92] One population of excitatory neurons in the entorhinal cortex that express *RORB* is also enriched for genes encoding axon-localized proteins and voltage-gated potassium channels [93]. One SST expression interneuron that is enriched with *NDFN*, *PROX1*, and *RELN* is associated with the early stage of AD in the frontal cortex [21]. SST-expressing interneurons in layer I and layer II are also affected in early AD in the middle temporal gyrus, whereas intratelencephalic excitatory neurons and parvalbumin-expressing interneurons disappear in later-stage AD [94]. Also, across brain regions study has revealed that Reelin signaling genes *RELN* and *DAB1*, kinase-associated genes *MAP2K5*, *PRKCA*, and *SPHKAP*, and some heparan sulfate proteoglycan biosynthesis genes are enriched in vulnerable excitatory neurons, while *RELN* and *DAB1* expression is also higher in vulnerable inhibitory neurons [95].

The brain atrophy is mainly driven by neuronal loss and synapse degeneration. Synaptic loss is strongly correlated with cognitive decline in both human and animal models of AD [96]. Indeed, evidence suggests that soluble forms of Aβ and tau species induce synaptotoxicity and propagate through neural circuits [97–99]. In addition, microglia and astrocytes can drive synaptic degeneration in animal models of aging and AD via ingestion of tagged synapses, contributing to cognitive decline [93, 100]. Despite its promise, the use of spatial transcriptomics (ST) to study vulnerable neurons in AD brains remains relatively limited.

Direct *in vivo* observations using STAR-MAP have enabled the simultaneous visualization of phosphorylated tau (p-tau) and microglia. In the TauPS2APP mouse model, p-tau signals were predominantly observed in the hippocampal region at 8 months and became more pronounced in both hippocampal and cortical regions by 13 months. Cell types and gene expression patterns were analyzed by the covariation of cell type composition versus p-tau density within 20 μm grided squares around p-tau staining. Oligodendrocytes were the most substantially enriched cell type near high p-tau signals in the corpus callosum and hippocampus. At 8 months, the majority of p-tau ^+^ neurons were CTX-Ex2 excitatory neurons, whereas at 13 months, the majority of p-tau ^+^ neurons were the CA1 excitatory neurons of the hippocampus. Inhibitory neurons account for < 20% of p-tau ^+ ^neurons, most of them were Pvalb neurons at 8 months, whereas at 13 months, most of them came from the Sst population. Furthermore, quantitative analysis revealed that p-tau signals were also enriched within a 10 μm radius of amyloid plaques. Given the relative depletion of neuronal cell bodies within this zone, the observed p-tau signals are likely attributable to dystrophic (injured) neurites that accumulate around plaques.

### Microglia in aging brains

Old age is associated with a decline in cognitive function and an increase in risk of neurodegenerative diseases like AD [101]. Over the past decade, concerted efforts have cataloged molecular and cellular hallmarks of aging that are conserved across different model systems [102–107]. Brain aging is a complex process accompanied by widespread cellular changes, yet how aged cells influence the brain environment, thereby contributing to overall tissue decline remains poorly understood [102]. Microglia, the long-lived, self-renewing cells responsible for phagocytic scavenging and immune surveillance, respond robustly to aging-related challenges and are considered to be important modifiers of brain aging [108–111].

Aging in the brain manifests as degeneration of both gray and white matter. White matter is composed of mostly myelinated axons that connect neurons from different brain regions and is critical for the integrity of functional circuits that correlate with cognitive impairment and an increasing risk of dementia [112]. Notably, aging-induced damage to myelinated nerve fibers is characterized by the release of lipid-rich, tightly compacted myelin debris that is difficult to clear. This demyelination process has also been implicated as a source of amyloid deposition. Myelin dysfunction causes the accumulation of the Aβ-producing machinery within axonal swellings and increases the cleavage of cortical amyloid precursor protein [113].

Transcriptomic analysis of sorted microglia from dissected white matter has revealed distinct signatures that are enriched for DAM and MHC Class II-related genes. These microglia tend to form clusters of 3–5 cells with large cell bodies and thick processes [114]. Similar transcriptomic signatures have been observed in microglia from cuprizone-treated demyelination and remyelination mouse models, where genes such as *Apoe*, *Axl*, *Igf1*, *Lyz2*, *Itgax*, *Gpnmb*, and *Apoc1* are upregulated during both phases [59]. Innovative approaches combining MERFISH with electron microscopy (EM) have further characterized the demyelination-associated microglia with a ‘foamy’ morphology that is located in the center of remyelination lesions in lysophosphatidylcholine (LPC)-induced WM injury [115]. The foamy microglia, laden with lipid droplets, expressed not only DAM signatures but also genes involved in lipid and cholesterol metabolism (e.g., *Plin2*, *Soat1*, *Abca1*, and *Abcg1*). In the lesions, foamy microglia are found in close proximity with interferon-responsive microglia (enriched with *Stat1*, *Ifit1*, *Usp18*, and *Rsad2*), oligodendrocytes, astrocytes and CD8 ^+^ T-cells (Fig. 3B).

Multiple studies using scRNAseq and ST have confirmed more changes in cell states, gene expression, and spatial organization of non-neuronal cells over neurons with age [85, 107, 116–119]. The corpus callosum, for instance, has proved to be one of the most impacted brain regions by aging [116, 117, 120]. Moreover, the third ventricle (V3) of the hypothalamus displays age-specific and cell-specific differential expressing genes [119]. Collectively, these studies reveal an enrichment of immune-modulatory and major histocompatibility complex class I (MHC-I)-mediated antigen presentation genes. Conversely, many neuron types show a decrease in expression of genes related to neuronal structure and function [107, 117, 119].

Microglia exhibited the largest number of genes that changed in expression with age [117, 118]. One study using a panel of MERFISH probes targeting 421 genes calculated an ‘activation score’ in the mouse frontal cortex and stratum of aging brains [116]. The activation scores were calculated based on selected gene expressions of microglia (B*2m*,* Trem2*,* Ccl2*,* Apoe*,* Axl*,* Itgax*,* Cd9*,* C1qa*,* C1qc*,* Lyz2*,* Ctss*) and Astrocytes (*C4b*,* C3*,* Serpina3n*,* Cxcl10*,* Gfap*,* Vim*,* Il18*, and *Hif3a)*. The data revealed that astrocytic and microglial clusters that are enriched in aged animals correlated with the inflammation level of adjacent oligodendrocytes. Another study using a 300 gene panel of MERFISH probes identified the ‘Spatial Ageing Clock’ of each cell in the brains of mice across the whole lifespan [118]. The ‘Spatial Ageing Clock’ predicts that microglia accelerate the aging of nearby cells. Applying spatial aging clocks to published ST data sets, the authors found that microglia, astrocytes, and oligodendrocytes exhibited accelerated aging in response to LPS [116]. Microglia also showed accelerated aging in AD in accordance with DAM enrichment [81]. In an experimental autoimmune encephalomyelitis (EAE) model of demyelination, microglia aging accelerated across the whole brain [121] while in localized demyelination injury, accelerated aging happened in all cell types around the injury [115].

In addition to glial cells, T cells, and Neural stem cells (NSC) exhibited the strongest age-related changes [85, 116, 119]. T cells increasingly infiltrate the brain with age, particularly in the corpus callosum and anterior commissure region [85, 118]. These T cells are hypothesized to exert a marked pro-aging proximity effect, as evident by the concomitant increase in IFNγ production and nearby cells showing a coordinate increase of IFNγ response gene expression (*Bst2*, *Stat1)* [118]. Conversely, NSCs have a strong pro-rejuvenating proximity effect on neighboring cells. NSCs were generally localized to the lateral ventricles throughout life and subsequently decreased with age [118, 119]. The neighboring cells of NSCs displayed increased expression of the ‘exocytosis’ marker as *Cd9*, which was confirmed by immunostaining.

Spatial mapping of the aging brain now provides unprecedented insights into cell proximity effects and intercellular interactions, offering a more comprehensive view of the molecular and cellular landscape during aging (Fig. 3B).

### AD in human samples

Experimental mouse models of AD are crucial for understanding disease mechanisms but studies have proved the limitations in fully representing human AD brain environment and molecular complexities [86, 122, 123]. Early foundational img-ST studies of human PIGs (Plaque-induced-Genes) revealed cell compositions within human amyloid plaque niche. The co-expression networks of PIGs and oligodendrocytes enriched genes show only similar alterations in human brain samples in the late stage of AD [79, 124]. Multiple studies have now begun to apply single-cell and spatial genomics to identify cellular vulnerabilities and molecular changes in human brains with AD [18, 20, 21, 23, 76, 86, 93, 125–131]. Studies in the human brain usually using post-mortem samples from brain banks, have given unprecedented insight into underlying pathophysiological mechanisms [132, 133]particularly to create a quantitative aggregate metric of the local burden of pathology that accompanies AD progression [134].

Recent studies have further refined our understanding of the human brain’s cytoarchitecture in the context of AD. For example, transcriptome-scale mapping in the dorsolateral prefrontal cortex has provided detailed insights into regional gene expression differences that may underpin cognitive decline [135]. In parallel, research focusing on vulnerable regions such as the middle temporal gyrus has identified key genes associated with selective vulnerability in AD [94, 136]. Comprehensive mapping of the neocortex and hypothalamus has revealed intricate cellular organizations and spatial-cellular patterns that not only delineate healthy brain architecture but also pinpoint alterations driven by disease [137].

More recently, advances in spatial resolution and analytic techniques have enabled comparisons between genetic and sporadic forms of AD [138]. Studies employing methods such as Stereo-seq and MERFISH have charted the prefrontal cortex and other brain regions in unprecedented detail, uncovering cell type-specific aging signatures and revealing how microglial activation and other immune responses contribute to amyloid-beta clearance [139]. Additionally, investigations into microglial mechanisms in immunized AD patients have highlighted the critical role of these cells in disease progression and potential therapeutic responses [140]. Collectively, these spatial transcriptomics studies are not only mapping the complex cellular interactions underlying AD pathology but also providing a blueprint for future diagnostic and treatment strategies by illuminating the molecular circuits that drive neurodegeneration.

## Conclusions

In conclusion, the application of spatial transcriptomics has significantly advanced our understanding of Alzheimer’s disease by revealing the intricate cellular architecture and dynamic interactions that underpin its progression. This review has highlighted how cutting-edge imaging and sequencing-based approaches have provided unprecedented insights into the spatial heterogeneity of key cell types, especially microglia, and their complex relationships with neurons, astrocytes, and oligodendrocytes in both aging and AD. Despite these promising advances, several challenges remain, such as achieving even higher resolution, integrating multi-omic datasets, and fully elucidating the molecular mechanisms driving cell-cell interactions in situ.

Future research that leverages these technological innovations is poised to deepen our mechanistic understanding of AD and identify novel therapeutic targets. For example, using deep tissue imaging together with ST to map comprehensive interaction cellular networks in a 3D brain [141, 142]. Incorporation with Raman scattering microscopy to identify metabolic changes at the cellular level of AD [143, 144]. By further dissecting the spatial interplay among brain cells, we can better appreciate how local microenvironments influence disease pathogenesis and progression. Furthermore, the determination of spatially resolved single-cell gene expression is the starting point to understanding the mechanisms by which cells achieve context-dependent phenotypes but doesn’t reveal the underlying transcriptional mechanisms. To achieve this goal, the parallel development of spatial epigenomics assays is required [145–149].

Moreover, studies have shown that following innate immune challenge, elevated levels of certain pro-inflammatory mRNAs do not necessarily correlate with their corresponding protein abundance, highlighting a dissociation between microglial mRNA and protein networks that is likely driven by post-transcriptional and translational regulation [150]. Notably, spatial proteomics technology has advanced rapidly, which benefit AD research greatly [151–153]particularly imaging-based protein-inclusive methods that multiplex the detection of 30–60 protein targets at subcellular resolution [154–158]. Additionally, mass spectrometry imaging (MSI) enables visualization of proteins and metabolites directly within tissues [159–161]. Direct visualization of Aβ proteoforms in the human brain revealed plaque heterogeneity, which was further confirmed by co-registration with histopathology [157, 162]. Examination of various tau isoforms identified an inverse correlation between synaptophysin and neurogranin levels and local phosphorylated tau (p-Tau) burden [158]. Additionally, multiple platforms have demonstrated microglial heterogeneity and identified plaque-associated microglial subsets, reinforcing the findings from spatial transcriptomics studies [154, 157]. Ultimately, a comprehensive understanding of these cellular network will pave the way for more effective, targeted interventions to combat AD and improve cognitive outcomes in the aging population.

## Data Availability

No datasets were generated or analysed during the current study.

## Abbreviations

Aβ: Amyloid beta
AD: Alzheimer’s disease
ALS: Amyotrophic lateral sclerosis
ARMs: Activated response microglia
BG: Basal ganglia
DAMs: Disease associated microglia
DBiT-seq: Deterministic Barcoding in Tissue for spatial omics sequencing
DG: Dentate gyrus
EM: Electron microscopy
FISH: Fluorescence in situ hybridization
FISSEQ: Fluorescent In Situ Sequencing
GWAS: Genome-wide association studies
IF: Immunofluorescence
IHC: Immunohistochemistry
Img-ST: Imaging-based spatial transcriptomic technology
ISS: In situ sequencing
MERFISH: Multiplexed Error-Robust FISH
MGnD: Microglial neurodegenerative phenotype
MS: Multiple sclerosis
MSI: Mass spectrometry imaging
NFT: Neurofibrillary tangles
NSC: Neural stem cells
PIGs: Plaque-induced genes
p-tau: Phosphorylated tau
RCA: Rolling circle amplification
scRNAseq: Single-cell RNA sequencing
Seq-ST: Sequencing-based spatial transcriptomic technology
seqFISH: Sequential fluorescence in situ hybridization
ST: Spatial transcriptomic technology
STARmap: Spatially Resolved Transcript Amplicon Readout Mapping
WM: White matters
